# Treatment of Secondary Syphilis; Action of Mercury; Anti-Mercurial Medication; Doses, Etc.

**Published:** 1848-09

**Authors:** 


*Treatment of Secondary Syphilis; Action of Mercury; Anti-Mercurial Medication; Doses, etc.* By M. Ricord.—I proceed with the consideration of secondary symptoms; and I would first direct your attention to the fact that the earlier manifestations are always less serious than the subsequent ones; and that the further we proceed along the links of secondary accidents, the more serious the prognosis becomes. But still, when the transitory and tertiary symptoms come on, they need not be looked upon with very great anxiety, except where no means have been used to arrest them. Iodine of potassium is all-powerful in controlling these affections, and in a very short time too. The particular seat of any syphilitic manifestation may add somewhat to the gravity of the case, particularly as refers to the tertiary forms, and it often happens that they leave after them indelible marks, and great deformity. Important functions may either be altered or entirely abolished by the destruction of certain organs; thus patients may get afflicted with deafness, dysphonia, aphonia, difficulty of deglutition, of pronunciation, &c. I need not insist any longer upon the prognosis of secondary symptoms; you see that they are powerfully influenced by a great variety of circumstances.

*Treatment of Secondary Syphilis.*—We have here, as well as in all other diseases, two indications to fulfil—first, to master the diathesis, and, secondly, to destroy the manifestations. As for the diathesis, I have repeatedly stated that it can be prevented but by the destruction of the chancre within the first five days of its existence; when this period is passed, we are never sure of preventing the general infection, and Hunter was mistaken when he thought he could arrest the chain of secondary accidents by his anti-syphilitic treatment. But I need not enter into this question again, I have sufficiently dilated upon it in speaking of primary sores.

*Treatment.*—There is hardly any remedy which has not been tried in this disease. Before I give you the list of them, it is but fair that you should know that secondary manifestations may disappear *suâ sponte*, without the intervention of any specific treatment. Among the means which have been employed, I may mention low diet, with the avoidance of much liquid; the same, with an abundance of fluids; either of which may be carried on for a longer or shorter period. Attention to diet is, in fact, extremely useful, and it ought to be particularly nutritious with individuals who suffer from debility. Antiphlogistic means are of great assistance, as long as they are not directed against the constitutional syphilis itself; but against local inflammations: they are then very useful. But whenever we have the mere secondary accidents to contend with, antiphlogistics should be avoided, for I am convinced by experiments that the blood in constitutional syphilis becomes very poor, so that it would be senseless to abstract any, and thereby increase the evil. Sudorifics had a great run at one time: guaiacum, sarsaparilla, sassafras, squills, &c., have by turns been extensively used; they may all be said to possess the same amount of efficacy—viz., very little. I generally give them to my patients when I wish to keep them under my observation, but they never arrest any of the accidents. They may, however be used as vehicles for other more useful substances. Purgatives are sometimes necessary to keep the primæ viæ free, but they possess no curative property. Among the number of remedies that have been employed, there are some which are well calculated to fulfil certain indications; they may be used as adjuvants, corrigents, &c. Opium is one of them. But the most powerful medication, the only one capable of keeping secondary syphilis in abeyance, is mercury. In order to watch the effects of this metal, we should first thoroughly understand its peculiar action, both in a pathogenic and therapeutic point of view. You all know that the action of mercury on the economy has been differently interpreted by divers observers; it has successively been looked upon as an excitant, stimulant, depressant, antiphlogistic, antiplastic, alterative, modifiant, &c. Some have maintained that it acts from one molecule to another—viz., that it gradually passes through the vessels containing the syphilitic virus, and carries it along with itself out of the system; above all, it is *anti-syphilitic.* Its pathogenic action is anything but constant. Some people are very easily brought under the influence of mercury, whilst others are quite refractory to it by whatever channel it may be introduced into the system. This metal, placed on an absorbing surface, may act directly on the same, and be absorbed a little while afterwards; or it may be absorbed without any local action whatsoever, and the local phenomena then occur *after* absorption. When applied to the skin, it may produce either eczema or erythema, but these eruptions have then no particular character, and the local irritation is an obstacle to absorption. These effects are observed as well on mucous membranes as on the skin. When the mercury is absorbed, it may produce stimulating effects, which bring forth a regular mercurial pyrexia: this is always to be looked upon as a very untoward circumstance, for the specific action is thereby very much impaired. When the mercury is absorbed, it sometimes re-acts on the channels through which it has passed; this effect is a sort of *contre-coup*. The first symptoms of the action of mercury on the system, are those of inflammation and increase of secretion, coinciding with a diminution of fibrine in the blood; but if the inflammation continue, a plastic re-action ensues, the fibrine, on the contrary, is relatively augmented, and the plasticity of the blood is heightened. Mercurial fever is generally followed by diarrhœa, resulting from a species of gastro-enteritis set up by the mercury; but the most common effect is mercurial stomatitis, or ptyalism.

*Symptoms.*—Patients, before they notice anything abnormal, complain of a metallic taste in the mouth; the breath is disagreeable, and even fœtid; the teeth feel uncomfortable, and are acutely sensitive; they give the patient the idea that they are longer; they lose their firmness; the gums seem to grasp them but feebly; the salivary secretion increases; the gums turn of a bright red, swell, and become very soft; the last molar or wisdom tooth, if it have come out, is the first to suffer. I must not forget to mention that these mercurial effects are intimately connected with the presence of the teeth, for stomatitis does not occur when the teeth are quite gone, or before they are cut. Wherever the least pressure is exercised there will be a certain tendency to the development of these symptoms, so that they will generally appear first on the side whereon the patient is in the habit of lying. As the affection advances, a greyish-blue, pseudo-membranous secretion takes place along the margin of the gums; the tumefaction of the latter goes on increasing; the swelled parts ulcerate; and this generally takes place first around the neck of the last molar. The internal surface of the cheeks retains the impress of the teeth on a level with the meeting of the upper and lower; the ulceration soon involves the cheeks themselves; the tongue swells, its sides are marked by indentations produced by the pressure of the teeth, and it may even share in the ulceration involving the neighbouring parts; indeed, it is not rare to see the cheeks, tongue, and gums, attacked by gangrene, and whilst all this is going on, the surrounding textures sympathize, and there is œdema of the cellular tissue and hypersecretion of the salivary glands. These are, however, not yet affected with inflammation; the abundant secretion is principally owing to irritation of the orifices of their ducts, and you should bear in mind that the inflammation does not primarily originate in the glands. Patients will sometimes eject a great quantity of a glairy, adhesive saliva, which at last comes to resemble diluted mud. If the inflammation is very violent it will react sympathetically upon the system, and general inflammatory phenomena become apparent. An experienced eye will never confound the mercurial effects with those of syphilis, but there are cases where both are combined in such a manner as to be distinguished with much difficulty. Mercurial ulcerations may, for instance, simulate mucous tubercles or patches. The only way to settle the question is, to suspend the administration of the mercury; the mercurial symptoms will then disappear, and the syphilitic ones remain and be easily recognised. The effects of mercury in regular doses are generally felt towards the beginning of the second week. Mercurial stomatitis cannot appear three or four months after the administration of the metal has been stopped, and those who still hold this as possible, have probably not sufficiently distinguished mercurial from scorbutic stomatitis. When you wish to increase the dose, you must watch the effect of moderate ones for eight or ten days; and you may fully believe me when I say, that the untoward or pathogenic effects of mercury are not the result of its prolonged administration, but that they arise principally from too large doses, continued for a week or two. The metal, the effects of which we are studying, may act on the nervous centres and produce what is called mercurial tremor, but this affection is very rare, when the mercury is given as an anti-syphilitic; you know that it is, on the contrary, very common with gilders and looking-glass makers, who are subjected to the influence of the metal in its vaporized form. Mercury may bring on apoplexy: I remember a very clear case illustrating this fact. The patient sank under the symptoms, and by the chemical analysis of the substance of the brain metallic mercury was discovered. Some people will tell you that mercury will bring on mental derangement: there is nothing very positive known on this head; but still, as its influence is well ascertained to extend sometimes to the brain, it might act upon the intellect by its effects on the encephalon. I am inclined to believe that this mercurial madness is perhaps only a sort of hydrargyrophobia, just as syphilitic alienation may merely be syphilophobia. Both hydrargyromania and syphilomania are, however, extremely rare. We have seen that certain mercurial manifestations, among which are eruptions, salivation, diarrhœa, &c., have generally appeared about eight or ten days after commencing use of mercury; but a long-continued mercurial course may impress upon the economy much deeper pathological alterations. These are of a slower growth, and present a series of quite different symptoms. Mercurial tremor, mercurial paralysis, and mercurial madness, belong to the latter category. When the system is fully under the influence of the metal, the syphilitic eruption may of course be modified by this circumstance; and we often see it in such cases assume more of the vesicular form, and cause a little pruritus, preserving, however, the general characters of a syphilitic eruption. If you should be so situated as to feel rather at a loss how to distinguish in a given case a mercurial from a syphilitic eruption, you will be greatly assisted by noticing the following contrasted characters:—When the two eruptions exist at the same time upon one individual, if you give up the mercury there is no tendency in the secondary eruption to fade away; on the contrary, it will increase in intensity; but the mercurial manifestations will diminish rapidly, and disappear, from the tenth to the fifteenth day. If, on the other hand, you were to keep on the mercury, you would see the mercurial eruption make rapid progress, whilst that resulting from syphilis would gradually disappear. I have several times tried this method, and found it answer remarkably well.

*Anti-mercurial medication.*—If you fine mercury doing mischief, I need hardly say that the first thing to be done is to leave it off, and thereby do away with the *origo mali.* Then the *primæ viæ* are to be attended to, and recourse be had to sulphur, either internally, or in the form of baths. This substance is very useful, provided there be no mercurial diarrhœa present. I generally give one drachm of sublimed sulphur, mixed with water, and one ounce of honey. I prescribe likewise, acid drinks, of which a very good one is the nitric acid lemonade, which seems to promote the plasticity of the blood. As to local applications, I know no better than hydrochloric acid, brushed, in a concentrated state, over the mercurial ulcerations. The cauterization is to be persisted in until it produces a sanguineous oozing. The pain is very intense, but of short duration, and the patients experience great relief immediately afterwards. The hydrochloric acid may also be given in the form of gargle: five ounces of lactuca sativa decoction, eighty minims of dilute hydrochloric acid, with about five ounces of honey, make a very excellent gargarism. When you cauterize with the concentrated acid, you must be careful to avoid the teeth, for it softens them very rapidly; and common cleanliness as regards the latter and the gums should be particularly enforced. When there is much diarrhœa, opium is indicated. I have always found it an excellent adjuvant and the best corrigent, and I cannot agree with those who have maintained that opium interferes with the specific action of mercury—the experience of every practitioner will at once settle the matter.

*Doses.*—The pathogenic accidents I have enumerated should always be prevented in the administration of mercury; indeed, there are few surgeons now-a-days who, in conformity with ancient customs, push it to profuse salivation, in the treatment of syphilitic diseases—most of them, like myself, only aim at its therapeutic action. The doses are strictly relative—the susceptibility of individuals should be studied, and the amount of mercury regulated thereby. We should always begin with a dose which is not likely to produce any unpleasant effect: this may be either one grain of the protoiodide of mercury, or one grain of the bichloride of the same metal, or the friction of one drachm of mercury ointment per day. The effect of these doses ought then to be watched: if they produce salivation, fever, or diarrhœa, the mercury must be stopped, and these complications removed. If no disturbance is produced by the doses I just named, they may be persisted in as long as we see the disease gradually receding and improving; but if the affection has not received any check after the first five or six days, the doses should be gradually increased, the results watched, and the remedy proceeded with as if the latter dose had been the one given at the outset.—*London Lancet.*

